# Functional Neurological Disorder in Saudi Arabia: An Update

**DOI:** 10.7759/cureus.47607

**Published:** 2023-10-24

**Authors:** Haythum O Tayeb

**Affiliations:** 1 Medicine, The Mind and Brain Studies Initiative, The Neuroscience Research Unit, Faculty of Medicine, King Abdulaziz University, Jeddah, SAU; 2 Neurology, Faculty of Medicine, King Abdulaziz University, Jeddah, SAU

**Keywords:** somatoform disorder, conversion disorder, psychogenic non-epilipetic seizures, functional neurological disorder, saudi arabia

## Abstract

Functional neurological disorder (FND) is characterized by neurological symptoms that lack congruence with traditional neurological diagnoses. Historically viewed through a Freudian psychoanalytic lens, FND has been conceptualized as a purely psychogenic disorder. However, the contemporary biopsychosocial perspective on FND emphasizes contributions of cognitive and neural circuit dysfunction and the disabling and involuntary nature of the illness. In Saudi Arabia, evidence suggests the prevalence of FND is significant. However, clinical programs and research focused on FND have been lacking. Studies from the region indicate that practitioners may have outdated views of FND. To address this, this narrative review provides an updated perspective on FND that is relevant to Saudi Arabia and the region. It delves into the evolving perception of FND, its underlying pathophysiology, risk factors, clinical presentations, and recent diagnostic and management advances. Unique features of FND in Saudi Arabia may include a significant role for family disputes as a risk factor, prevalent supernatural perceptions of FND, high prevalence of somatization, and cognitive dysfunction, and a potential favorable prognosis. The article concludes by providing the following recommendations related to FND in Saudi Arabia and the region: i) building educational programs to update clinicians about contemporary biopsychosocial perspectives on FND; ii) emphasizing a positive diagnostic approach based on clinical findings in FND; iii) instituting multidisciplinary programs to care for FND patients; iv) supporting systematic research efforts to explore culture-specific FND risk factors, patient outcome measures, and attitudes toward the disorder; v) developing national FND clinical practice guidelines; and vi) launching awareness campaigns to reduce FND stigma.

## Introduction and background

Functional neurological disorder (FND) is a neuropsychiatric disorder characterized by neurological symptoms that are incongruent with neurological diagnoses [1]. Also known as conversion disorder, FND is a common somatoform disorder affecting the lives of millions of individuals. The prevalence of the disorder is estimated to be around 10%, but it is likely that this is an underestimate as the disorder is likely underdiagnosed [2]. FND symptoms are associated with significant disability, high utilization of healthcare services, and poor quality of life [3]. Traditionally, the disorder has been viewed from a Freudian psychoanalytic perspective. The neurological symptoms seen in FND have been ascribed to maladaptive ego defense mechanisms, wherein individuals shield themselves from psychic conflicts by converting them into neurological symptoms [4]. According to this perspective, FND is a psychogenic disorder that must be treated with psychoanalysis [4]. In contrast, the contemporary perspective on FND embraces accumulating recent evidence indicating the presence of cognitive and neural circuit dysfunction in patients with FND. In this light, FND is viewed as a biopsychosocial disorder that is optimally managed through a multidisciplinary approach, rehabilitative treatments, as well as psychotherapy and pharmacotherapy [5].

FND has garnered considerable attention from clinicians and researchers over the past decade, leading to advancements in the understanding and management of the disorder [5]. This wave of focus on FND research and clinical care has not made a significant impact in the Middle Eastern Arab countries and literature on FND from this region remains limited. In Saudi Arabia, evidence suggests the prevalence of somatoform and related disorders is at least on par with the rest of the world, ranging between 5 and 40% [6-9]. Studies from the region suggest practitioners' views on FND may be outdated, and practitioners may be unfamiliar with the contemporary perspective on FND and the potential specific aspects of the disorder in the region [7,10].

This narrative review aims to provide an updated practical perspective on FND that is relevant to Saudi Arabia and the Arab Middle East. The aim is to describe how the perception of FND has evolved, its pathophysiologic underpinnings, risk factors, clinical profile, and recent advances in how it is diagnosed and managed. This paper examines the landscape of FND research in Saudi Arabia and concludes by providing recommendations regarding how FND programs and research may be developed in the region.

## Review

History of FND: from the antiquities to the Diagnostic and Statistical Manual of Mental Disorders (DSM)

Medical and cultural interpretations of paroxysmal neurological symptoms have evolved over time [11]. In prehistoric times, in the absence of medical knowledge, unexplained medical phenomena were often attributed to supernatural forces. Convulsions, paralysis, and altered states of consciousness were often seen as evidence of possession by spirits, curses, malevolent sorcery, or divine intervention [11]. In Saudi Arabia and the Middle East (as in many other world regions), some of these erroneous and supernatural perceptions of FND persist until today [12-14]. In the 19th century, Charcot’s work advanced the conceptualization that FND is a brain and mind disorder, emphasizing the role of stress, emotional dysregulation, and psychopathology. Charcot’s disciple, Sigmund Freud, coined the concept of conversion disorder, describing how psychological trauma or distress could be "converted" by unconscious maladaptive defense mechanisms into physical symptoms that may be easier to understand and accept [4]. Freud’s psychodynamic treatment approach emphasized that affected individuals must re-experience traumatic events repressed out of consciousness and reformulate a more adaptive response to them. The terms primary and secondary gains emerged. Primary gains from FND symptoms refer to direct psychological relief or satisfaction obtained because of the converted symptoms, whereas secondary gains are indirect gains resulting from the reactions of those around the person or the person’s environment. In that era, the practice of neurology and psychiatry diverged, and FND was conceptualized as a “psychogenic disorder”, with diagnosis and therapy relying heavily on the psychoanalytic perspective on the disorder [11]. It is now acknowledged that the psychoanalytic perspective is insufficient to explain the pathophysiology of FND and that overemphasis on trauma and gains may be associated with stigma [15,16].

The concept of FND has subsequently evolved with the emergence and evolution of the DSM [17]. In the earlier versions of the DSM, conversion and dissociative reactions were conceptualized as neuroses. The DSM-I referred to “conversion reactions” under the category of “psychoneurotic disorders”. The DSM-II introduced "Hysterical Neurosis, Conversion Type". The DSM-III moved away from linking conversion and dissociation and adhered to the appearance of somatic symptoms as criteria, fitting conversion disorder under the newly created category of “Somatoform Disorders”. The DSM IV eliminated references to hysterical neurosis, and defined conversion disorder by the presence of neurologic symptoms that are inconsistent with neuropathophysiologic explanations. The DSM V abandoned the term conversion, recognizing that this concept lacks empirical evidence and that the psychogenic origin for FND symptoms is not always clinically demonstrable [17]. In addition, this iteration no longer required the presence of an identifiable traumatic or stressful event. As such, with successive versions of the DSM, the psychiatric community progressively moved away from a pure psychoanalytic perspective and toward an etiologically agnostic stance. This change reflects a paradigm shift in the understanding of FND, with a focus on the involuntary and disabling nature of the disorder. This reconceptualization acknowledges accumulating evidence that FND is a complex disorder with an interplay of psychopathology, sociocultural factors, stress, and neural network dysfunction [15].

Contemporary perceptions of FND: neural circuit dysfunction and the biopsychosocial perspective

**Figure 1:**
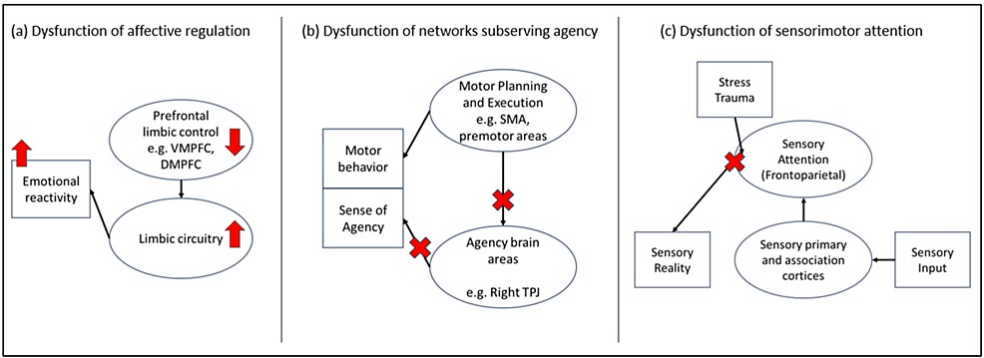
Diagrammatic illustration of notable examples of neural network dysfunction in FND VMPFC: Ventromedial prefrontal cortex, DMPFC: dorsomedial prefrontal cortex, SMA: supplementary motor area, TPJ: temporoparietal junction, FND: functional neurological disorder. Upward arrow: Increased activity, downward arrow: decreased activity, X: lesion observed in FND.

Although the pathophysiology of FND is far from being adequately understood, evidence from a large body of neuroscience research suggests that the deficits seen in FND involve dysfunction in neural networks. For excellent reviews on this, the reader is encouraged to read the reviews by Drane et al. and Milano et al. [18,19]. Figure 1 illustrates diagrammatically several notable examples of neural circuit dysfunction in FND.

An integral component of FND’s neural circuit pathology is the disturbance circuitry of affective regulation. There is evidence of increased activation of the amygdala and periaqueductal gray and dysregulated prefrontal top-down control of these limbic structures. This may explain increased and sustained emotional reactivity in FND. Furthermore, limbic and motor control circuits appear to have increased functional connectivity, explaining how involuntary movements or weakness may occur. Another notable dysfunctional neural circuit in FND is that of self-agency, a person’s sense of ownership of their own movements and perceptions. After a movement is performed, feedback signals travel from sensorimotor areas to multimodal sensory areas (including the right temporoparietal junction) to generate the sense of self-agency of that movement. Studies have shown right temporoparietal dysfunction in patients with motor FND, leading to a lack of ownership of performed movements [18]. Other demonstrable neural circuit abnormalities in FND include dysregulation of the fronto-parietal attentional network, which subserves sensory processing and integration. Its dysfunction may lead to erroneous overemphasis, neglect, or improper integration of sensory experiences. Stressful or traumatic experiences (such as childhood trauma, social adversity, chronic illnesses, and others) have the potential to alter this circuit, leading to improper integration of sensory experiences, and generation of inaccurate sensorimotor predictions. As a result, the brain’s response to stressful external events, thoughts, or bodily changes may be exaggerated, neglected, or inappropriate [18].

The nature of neural network dysfunction in each patient is thought to dictate the emergent FND symptomatology, thus creating the FND core symptom subtypes. FND symptoms may therefore be neurologically localizable to the dysfunctional circuits, even if lesions are not seen in routine neuroimaging or clinical investigations [18,19].

Clinical features: the era of positive diagnosis

FND may present acutely, chronically, or in a relapsing manner. An inciting physical or psychological traumatic event may take place but is not required for the diagnosis [17]. There is a spectrum of FND symptoms and patients may have one or more functional symptoms together or separately during their illness. However, one core symptom usually predominates. Core FND subtypes include functional weakness (motor FND), psychogenic nonepileptic seizures (PNESs), functional movement disorders, functional sensory loss, functional visual loss, functional speech disturbances, and functional cognitive dysfunction [1,15].

Functional weakness often presents as hemiparesis or quadriparesis but may affect one limb or both legs. PNES presents with seizure-like events, characterized by altered awareness and focal or generalized limb shaking without associated epileptiform EEG changes. Functional abnormal movements may present with assimilating tremors, gait unsteadiness, slowing of movement, sudden jerks, and dystonia-like posturing. Functional speech abnormalities include hoarseness slurred speech, stuttering, or speaking with a foreign language or accent. Functional sensory loss may affect any body part and does not align with known sensory pathways. Hemianesthesia that abruptly stops at the midline (midline splitting) is typical. Functional visual disturbances present as intermittent blurred vision, double vision, or eye movement abnormalities. Functional cognitive dysfunction may present with memory issues, word-finding difficulties, and mental ‘fogging’. Severe memory loss and fugue states may be seen. These symptoms are distinguished from cognitive symptoms accompanying mood disorders and anxiety by the loss of insight in the former and its presence in the latter [1,17].

In addition to the main functional symptoms, patients may display other characteristic symptoms or signs. Patients may exhibit La belle indifference, a lack of concern incongruous with the symptom, and severity and disability. Unexplained pain syndromes are commonly seen and should raise the possibility of FND in patients with motor FND or functional movement disorders [1,2]. In addition, FND patients often report numerous physical symptoms like abdominal pains, intractable muscle and body pain, and fatigue. Psychiatric symptoms and diagnoses are also seen but are not characteristic or required for the diagnosis. Patients may report dissociative symptoms, including being in a dream-like state, derealization, depersonalization, and out-of-body experiences. Borderline, histrionic, and narcissistic personality disorders may be present. Past history of childhood abuse or neglect is common but not invariably present in patients with FND [2,15,17,20].

Studies suggest that the symptomatology of FND in Saudi Arabia and the Middle East may have some unique features, although studies are insufficient to draw firm conclusions. Although core FND symptoms likely share more similarities than differences across cultures, region-specific features, and culture-bound syndromes have been described [19,21,22]. This is because FND symptoms partly flow from the culture-dependent models encoded in the brain of how individuals may behave in health and disease [23]. In Saudi Arabia, small studies surveying how FND manifests have been reported. In a small unpublished retrospective study, the author observed that PNES was the most common core FND subtype, followed by functional abnormal movements and sensory FND. Cognitive symptoms were frequent among this sample and may be associated with negative patient outcomes. Previous cross-sectional studies from Saudi Arabia, Oman, and Qatar found higher rates of somatization than FND and demonstrated a high prevalence of pain, sensory symptoms, and fatigue [6-9,24,25]. In neighboring Iran, PNES was also found to be the most common FND subtype, and the severity of symptoms was found to be higher than a comparison group from South America. The authors suggested that local social restrictions may contribute to this finding [21,22].

Although the diagnosis of FND requires careful consideration of neurological diagnoses, FND should not merely be thought of as a diagnosis of exclusion. Positive clinical findings consistent with FND should be sought and confirmed. Table 1 summarizes key clinical findings that are associated with excellent specificity that can help rule in the diagnosis of FND. For example, anatomical inconsistency, the Hoover and abductor signs, and give-way weakness were found to have specificity values of 86-100%. If a patient’s history fits with the clinical profile of FND, and physical examination demonstrates some of the findings listed in Table 1, clinicians should be able to directly make the FND diagnosis. If specific neurologic differential diagnoses are being considered, a targeted workup may be performed to aid in the diagnostic process but not to diagnose FND. Incidental findings on unnecessary tests may derail the diagnostic process and instill doubt in the minds of patients [1,2].

**Table 1:** Key clinical signs allowing a positive FND diagnosis FND: Functional neurological disorder

Sign	Description
Inconsistency with anatomical pathways	When patients present with unlocalizable neurological symptoms and signs. E.g. weakness with a distribution that does not respect anatomical pyramidal, root or nerve supply.
Splitting the midline	When sensory loss stops exactly at the midline; when vibration applied to the frontal skull bones is not felt at all across the midline.
Absent pronator drift	Downward drift of outstretched arm without pronation.
Variability	Fluctuating in a non-neurological manner. E.g. worsening when observed; resolving when unobserved; worsening with attention to involved parts.
Distractibility	Symptom resolution when a patient is distracted. Example: Unilateral tremors stop when examiner gets patient to focus on the contralateral hand.
Suggestibility	Symptom changes as a function of the examiner’s suggestions. Example: Tuning fork elicits a functional seizure or abnormal movements.
Hoover sign	Resolution of unilateral hip extension weakness when the contralateral hip is flexed against resistance.
Hip abductor sign	Resolution of unilateral hip abduction weakness with contralateral hip abduction against resistance.
Finger abduction sign	Resolution of unilateral finger abduction weakness with contralateral finger abduction against resistance.
Give-way weakness	Sudden loss of strength in the midst of power examination against resistance.
Co-contraction	Concurrent contraction of agonist and antagonist muscles during weakness or abnormal movements.
Whack-a-mole sign	Relocation of abnormal movement in a body part upon suppression by the examiner to another distant body part.
Uneconomic posturing	Adopting difficult demanding postures. E.g. walking as if the ground is slippery or shaky; swaying to maintain balance.
Functional facial spasm	Pulling of the angle of the lip with excessive platysma activation.
Convergence spasm	Excessive convergence especially when focused on near objects, associated with pain and blurred vision or diplopia.

Diagnostic criteria for FND syndromes have been proposed and are helpful in guiding the diagnostic process [26,27]. PNES diagnostic criteria take advantage of the sensitivity of the video EEG in demonstrating the lack of electrographic seizures during PNES events. These criteria also incorporate video-recorded semiologic features of the events [27]. Some of these features include forced eye closure during convulsions, intact awareness during bilateral convulsions, crying during convulsions, a start-stop pattern, and others [27]. The Fahn-Williams and other diagnostic criteria for psychogenic movement disorders specify levels of diagnostic certainty (possible, probable, clinically definite) based on incongruence or inconsistency of the movement disorder, with response to suggestion, placebo, or psychotherapy defining the highest level of diagnostic certainty [26]. Although the diagnostic performance of these positive clinical findings and criteria has been well documented, there may be a lag in clinicians’ adoption of the positive diagnostic approach and a persistent reliance on framing FND as a diagnosis of exclusion. This may lead to delays in diagnosis and negatively impact the certainty and quality with which FND diagnoses are delivered by clinicians and accepted by the patients [5,28].

Management of FND: the multidisciplinary approach

The initial and crucial step in managing FND is the proper and accurate presentation of the diagnosis to the patient, followed by counseling and education. Proper counseling not only establishes the therapeutic alliance with the patient but may also facilitate acceptance of the diagnosis and directly lead to short-term improvements in FND symptoms. The first essential element when presenting the diagnosis is to avoid accusing patients of faking their symptoms or blaming them for their inability to control these symptoms. The best approach is to explain FND symptoms from a biopsychosocial perspective, highlighting how the brain’s normal functioning may be disrupted by psychosocial stressors, maladaptive coping mechanisms, neurodevelopmental factors, and trauma. The approach of incorporating neurobiological aspects of the disorders’ pathophysiology may reduce the likelihood of rejecting the diagnosis and improve their participation in the treatment plan [28-30].

Clinicians should then consider the role of psychotherapy, particularly if patients do not improve upon follow-up after the initial diagnosis presentation. Cognitive-behavioral therapy (CBT) was shown in clinical trials to improve FND outcomes [31-33]. CBT helps patients examine their automatic thoughts about their symptoms and change the behaviors that flow out of these thoughts. In patients with episodic symptoms, specific CBT techniques are used to enable patients to recognize premonitory autonomic or somatic symptoms that may precede their episodes. Mindfulness-based psychotherapies may also help patients with FND gain awareness of their emotional and bodily responses to relevant stimuli [34]. Other forms of therapy, including psychodynamic psychotherapy and trauma therapies may also be of help [31] and it may be helpful to consult with a neuropsychiatrist or a psychologist to determine the most fitting mode of psychotherapy for a given patient.

Psychologically minded rehabilitation approaches have proven to be a useful component of multidisciplinary care in FND [35,36]. Physical therapy may help patients with motor FND or functional movements. Patients may benefit from techniques that alter how they attend to affected limbs. Examples include distraction techniques and superimposition of unimpaired movements on functional involuntary movements [36]. During occupational therapy, focus is shifted from impairment to daily functional activity goals, and aids like wheelchairs and canes are utilized only as needed. For FND patients with speech symptoms, speech and language therapy may be helpful. International consensus recommendations are available for physical, occupational, and speech therapy in FND and may serve as useful guidance for clinicians.

Other treatments may be considered in FND. Pharmacotherapy may be helpful in treating comorbid symptoms of FND [37]. Antidepressants and anxiolytics may help treat depression and anxiety symptoms. When pain is present tricyclic antidepressants, duloxetine, and pregabalin may be helpful. There is little evidence to support the use of pharmacotherapy in FND. It has been shown in small clinical trials that SSRIs may help reduce the frequency of PNES events [38]. Other than that, the evidence does not support the routine use of pharmacotherapy in FND. There is limited evidence to back hypnosis, transcranial magnetic stimulation, and conscious sedation in FND, and they should not be routinely considered [39]. Of note, discontinuing therapies that were started for previously erroneously diagnosed neurological conditions may be important. For example, antiseizure medications should typically be discontinued in patients with PNES. Discontinuing these unnecessary therapies avoids their side effects, eliminates uncertainty about the FND diagnosis, and focuses the patient and treating team’s efforts on FND-specific therapies [1].

Because optimal diagnosis and management of FND often involve multiple healthcare providers from different disciplines, it is crucial that these professionals work together as a team. The team may include professionals from neurology, psychiatry, psychology, physical therapy, occupational therapy, speech and language therapy, social work, and others. Multidisciplinary care ensures a seamless patient journey, reduces inconsistent messages from different professionals, and has been shown to be associated with improvement in physical and psychological symptoms, and shorter hospitalization [5,15,37].

FND in Saudi Arabia: insights and recommendations

Although systematic epidemiological studies focused on FND in Saudi Arabia and the region are lacking, FND appears to be prevalent in Saudi Arabia. The author has observed in an unpublished retrospective chart review that about 10% of patients seen at an academic neuropsychiatry clinic in Saudi Arabia had FND, a prevalence equivalent to that seen in other parts of the world [2]. Cross-sectional studies surveying primary care patients for somatization found a prevalence of up to 40% [6,7]. Due to the paucity of systematic large-scale research about FND in Arab Middle Eastern countries, our knowledge about psychosocial factors, the impact of cultural beliefs, stigma, and the spectrum and quality of healthcare services available to these patients stems from small studies and from studies of other (mostly Western) patient populations. This knowledge gap is important for planning FND programs in this region. In addition, it is conceivable that there may be region-specific differences from the West in how FND manifests [40], and gaining a deeper understanding of these region-specific or culture-specific variations may contribute to an improved overall understanding of the disorder itself. Therefore, there is an imperative need to enhance our understanding of FND in this region and improve clinical and research FND programs. Table 2 outlines the author's key recommendations to achieve these goals.

**Table 2:** Key recommendations to advance FND clinical care and research in Saudi Arabia FND: Functional neurological disorder

Key recommendations to advance FND clinical care and research in Saudi Arabia
1. Building educational programs to update clinicians and educate trainees about contemporary biopsychosocial perspectives on FND
2. Emphasizing a positive diagnostic approach based on clinical findings in FND
3. Instituting multidisciplinary programs to care for FND patients, including training and recruitment of professionals from neurology, psychiatry, psychology, family medicine, physical therapy, occupational therapy, and speech therapy.
4. Supporting systematic research efforts to explore culture-specific FND risk factors, patient outcome measures, and clinician attitudes toward the disorder
5. Developing and implementing national FND clinical practice guidelines
6. Launching awareness campaigns to reduce FND stigma

The literature suggests the possibility of unique culture-specific features of FND in Saudi Arabia. First, the risk factor profile may be different or incompletely understood. In our yet unpublished retrospective chart review of FND, family disputes were the most common psychosocial risk factors, and sexual abuse was reported by 15% of patients. Similar findings were reported in an Iranian sample of FND patients [21,41]. In prior studies of Saudi patients with somatization, social stressors, educational difficulties, and other stressors were emphasized [6,8,9]. Therefore, the prevalence of sexual abuse may be lower than that observed in Western patient populations, and family disputes may be a more important risk factor in a hierarchical society where a struggle between modernity and conservatism continues to unfold. The nature of family disputes and psychosocial stressors contributing to FND in this region merit exploration in further studies. It must also be noted that sexual abuse may be underreported in this patient population and that some of the family disputes may be related to instances of domestic or sexual abuse [22].

There may also be unique features in the clinical symptomatology of FND in Saudi Arabia but studies are insufficient. In the author’s unpublished retrospective study, cognitive dysfunction was prevalent and may have been associated with negative outcomes upon follow-up. Patients in this sample reported a high prevalence of supernatural explanations of FND symptoms. Reported supernatural themes included possession, sorcery, divine punishment, and others. These beliefs may have clinical implications and may be associated with stigma [12,42]. Somatic symptoms, pain, and anxiety also seem to be particularly prevalent in Saudi patients with somatoform disorder, and somatization was more commonly observed than FND [6-9]. Previous cross-sectional studies from the region also found higher rates of somatization than FND [6-9,24,25]. This may be because specific FND criteria were not systematically used in clinical practice where these studies were done, but there may be a distinct profile of somatoform psychopathology in this region where psychosocial stressors are more likely to be expressed through somatization rather than repression and dissociation. This may relate to the fact that it is culturally quite acceptable in Saudi Arabia that females may express psychological distress as somatic complaints [9]. Of course, these conjectures remain observational and require systematic empirical studies.

Another intriguing point of interest is how cultural and religious beliefs may influence patient acceptance and response to FND. Based on religious culture, Saudis invariably believe that the soul (a mysterious spiritual entity) can interact with and influence the body. This belief is based on mind-body dualism, the philosophical stance that the mind is distinct from but can interact with the brain and the body. This philosophical stance was shown to be common in Saudi Arabia and may be associated with the stigma of mental and neurological disorders [12,43]. However, this belief may also facilitate acceptance of bodily symptoms resulting from distress in psychological and mental distress. It may be worthwhile exploring how these concepts relate to FND and how they may be incorporated when counseling and educating Saudi patients with FND. A balanced discussion of the nature of the human mind and the relationship between the brain and body may be helpful in facilitating acceptance of the diagnosis [44-46]. Indeed, exploration of themes related to faith and culture may prove crucial when counseling patients with FND from this world region [47].

In addition, the attitudes of clinicians dealing with FND in Saudi Arabia are worthy of exploration. In unpublished research, the author and colleagues studied the attitudes of a sample of Saudi neurologists and psychiatrists and found that nearly a third of participants endorsed a predominantly psychogenic view of the disorder, with underemphasis on the disabling and involuntary nature of the disorder, and the neural network dysfunction that is now known to be part and parcel of FND. In this sample, this psychogenic perspective on FND was associated with reduced willingness to participate in multidisciplinary FND care. These attitudes may alter patient outcomes [28-30].

Finally, to the authors’ knowledge, there are no formal, multidisciplinary specialized FND services in Saudi Arabia. In addition, there are no clinical practice guidelines that guide clinicians and institutions when it comes to how to deliver FND care. There may be a wide variation in practice patterns among institutions and individual healthcare professionals taking care of FND [48]. In the author’s experience and based on small previous studies, when patients with FND in Saudi Arabia present to emergency rooms, neurology, psychiatry, or primary care offices, the diagnosis may be presented in an abbreviated or incomplete manner, with emphasis on ruling out neurological and psychiatric diagnoses [7,8,48]. This observation is worthy of future systematic research. In an unpublished retrospective study, the author observed favorable patient outcomes when the diagnosis was explained by a neuropsychiatrist. Unfortunately, it is not uncommon that patients are given unclear instructions or limited options with poor access to appointments. Despite the estimated prevalence of related disorders in the country, the proportion of somatoform disorders among patients referred to psychiatry from primary healthcare centers and general hospitals was only 10% [8]. While two-thirds of FND patients received pharmacotherapy, psychotherapy was only offered to less than 4% of patients [7]. In the author’s experience, Saudi patients with FND struggle to find healthcare professionals who are qualified, available, and interested in providing FND care. Many consider traveling abroad, teleconsultations, or unideal options. Suboptimal FND care is not specific to Saudi Arabia and was reported elsewhere in the world [16,30,41,49].

## Conclusions

In conclusion, FND is a prevalent problem worldwide. Although large-scale epidemiologic research is lacking, available several small studies suggest that FND is prevalent in Saudi Arabia. FND has not received a proportionate degree of focus from clinicians and healthcare systems in Saudi Arabia. FND management hinges upon proper diagnosis presentation and effective multidisciplinary care delivered by a compassionate healthcare team. These aspects may need to be emphasized when planning and providing FND care in Saudi Arabia. There may be unique features in how Saudi FND patients present and how they may perceive and deal with the disorder.

The field of FND has moved rapidly over the course of the past couple of decades. This review article presented vital recommendations and a roadmap to improve FND clinical services and research in Saudi Arabia and the Middle East Arab countries, and many of these recommendations would apply at a global level as well. Examples of sorely needed FND research programs include epidemiological research to study the prevalence and risk factors of the disorder, neuroscience research to elucidate potential biomarkers, and clinical research to validate FND diagnostic tools and outcome measures, as well as research looking to find the best psychotherapeutic and counseling approaches to FND in Saudi Arabia. There is a pressing need for developing, disseminating, and implementing training programs and clinical practice guidelines. Substantial efforts, resources, and leadership are needed to achieve these goals in Saudi Arabia and worldwide.

## References

[1] Aybek S, Perez DL (2022). Diagnosis and management of functional neurological disorder. BMJ.

[2] Bennett K, Diamond C, Hoeritzauer I, Gardiner P, McWhirter L, Carson A, Stone J (2021). A practical review of functional neurological disorder (FND) for the general physician. Clin Med (Lond).

[3] Gelauff J, Stone J, Edwards M, Carson A (2014). The prognosis of functional (psychogenic) motor symptoms: a systematic review. J Neurol Neurosurg Psychiatry.

[4] Kanaan RA (2016). Chapter 4 - Freud's hysteria and its legacy. Handbook of Clinical Neurology.

[5] Perez DL, Edwards MJ, Nielsen G, Kozlowska K, Hallett M, LaFrance WC Jr (2021). Decade of progress in motor functional neurological disorder: continuing the momentum. J Neurol Neurosurg Psychiatry.

[6] Becker S, Al Zaid K, Al Faris E (2002). Screening for somatization and depression in Saudi Arabia: a validation study of the PHQ in primary care. Int J Psychiatry Med.

[7] Alanazi AO, Aljohani RA, Aljohani MF (2021). Epidemiological features and clinical manifestations of patients with somatoform disorder at a tertiary medical city in Riyadh, Saudi Arabia. Cureus.

[8] Qureshi NA, Al Habeeb TA, Al Ghamdy YS, Magzoub ME, Van der Molen HT (2001). Psychiatric co-morbidity in primary care and hospital referrals, Saudi Arabia. East Mediterr Health J.

[9] Alsaleem M, Ghazwani A (2014). Screening for somatoform disorders among adult patients attending primary health care centers. Al-Azhar Assiut Med J.

[10] Alamrawy RG, Abdel Tawab AM, Omran HA (2023). Unveiling the enigma: physicians’ perceptions of functional neurological disorders in Egypt—a cross-sectional study. Egypt J Neurol Psychiatry Neurosurg.

[11] Raynor G, Baslet G (2021). A historical review of functional neurological disorder and comparison to contemporary models. Epilepsy Behav Rep.

[12] Tayeb HO (2019). Epilepsy stigma in Saudi Arabia: the roles of mind-body dualism, supernatural beliefs, and religiosity. Epilepsy Behav.

[13] Obeid T, Abulaban A, Al-Ghatani F, Al-Malki AR, Al-Ghamdi A (2012). Possession by 'Jinn' as a cause of epilepsy (Saraa): a study from Saudi Arabia. Seizure.

[14] Sawant NS, Umate MS (2022). Clinical manifestations and cultural correlates of psychogenic nonepileptic seizure symptoms: an Indian perspective. J Family Med Prim Care.

[15] Perez DL, Aybek S, Nicholson TR, Kozlowska K, Arciniegas DB, LaFrance WC Jr (2020). Functional neurological (conversion) disorder: a core neuropsychiatric disorder. J Neuropsychiatry Clin Neurosci.

[16] Rommelfanger KS, Factor SA, LaRoche S, Rosen P, Young R, Rapaport MH (2017). Disentangling stigma from functional neurological disorders: conference report and roadmap for the future. Front Neurol.

[17] Levenson JL, Sharpe M (2016). Chapter 16 - the classification of conversion disorder (functional neurologic symptom disorder) in ICD and DSM. Handbook of Clinical Neurology.

[18] Drane DL, Fani N, Hallett M, Khalsa SS, Perez DL, Roberts NA (2020). A framework for understanding the pathophysiology of functional neurological disorder. CNS Spectr.

[19] Milano BA, Moutoussis M, Convertino L (2023). The neurobiology of functional neurological disorders characterised by impaired awareness. Front Psychiatry.

[20] Myers L, Trobliger R, Bortnik K, Lancman M (2018). Are there gender differences in those diagnosed with psychogenic nonepileptic seizures?. Epilepsy Behav.

[21] Asadi-Pooya AA, AlBaradie R, Sawchuk T, Bahrami Z, Al Amer A, Buchhalter J (2019). Psychogenic nonepileptic seizures in children and adolescents: an international cross-cultural study. Epilepsy Behav.

[22] Asadi-Pooya AA, Valente K, Alessi R, Tinker J (2017). Semiology of psychogenic nonepileptic seizures: an international cross-cultural study. Epilepsy Behav.

[23] Canna M, Seligman R (2020). Dealing with the unknown. Functional neurological disorder (FND) and the conversion of cultural meaning. Soc Sci Med.

[24] Abdullatif J, Certal V, Zaghi S, Song SA, Chang ET, Gillespie MB, Camacho M (2016). Maxillary expansion and maxillomandibular expansion for adult OSA: a systematic review and meta-analysis. J Craniomaxillofac Surg.

[25] Bener A, Ghuloum S, Al-Mulla AA, Al-Marri S, Hashim MS, Elbagi IE (2010). Prevalence of somatisation and psychologisation among patients visiting primary health care centres in the State of Qatar. Libyan J Med.

[26] Yoshida K (2020). Clinical characteristics of functional movement disorders in the Stomatognathic system. Front Neurol.

[27] LaFrance WC Jr, Baker GA, Duncan R, Goldstein LH, Reuber M (2013). Minimum requirements for the diagnosis of psychogenic nonepileptic seizures: a staged approach: a report from the International League Against Epilepsy Nonepileptic Seizures Task Force. Epilepsia.

[28] Stone J, Wojcik W, Durrance D (2002). What should we say to patients with symptoms unexplained by disease? The "number needed to offend". BMJ.

[29] Edwards MJ, Yogarajah M, Stone J (2023). Why functional neurological disorder is not feigning or malingering. Nat Rev Neurol.

[30] Hustvedt S (2013). Philosophy matters in brain matters. Seizure.

[31] Gutkin M, McLean L, Brown R, Kanaan RA (2020). Systematic review of psychotherapy for adults with functional neurological disorder. J Neurol Neurosurg Psychiatry.

[32] O'Connell N, Watson G, Grey C, Pastena R, McKeown K, David AS (2020). Outpatient CBT for motor functional neurological disorder and other neuropsychiatric conditions: a retrospective case comparison. J Neuropsychiatry Clin Neurosci.

[33] O'Neal MA, Baslet G (2018). Treatment for patients with a functional neurological disorder (conversion disorder): an integrated approach. Am J Psychiatry.

[34] Baslet G, Ridlon R, Raynor G, Gonsalvez I, Dworetzky BA (2022). Sustained improvement with mindfulness-based therapy for psychogenic nonepileptic seizures. Epilepsy Behav.

[35] Nicholson C, Edwards MJ, Carson AJ (2020). Occupational therapy consensus recommendations for functional neurological disorder. J Neurol Neurosurg Psychiatry.

[36] Kasia K, Nicola G, Stephen S, Blanche S (2021). Psychologically informed physiotherapy as part of a multidisciplinary rehabilitation program for children and adolescents with functional neurological disorder: physical and mental health outcomes. J Paediatr Child Health.

[37] Perjoc RS, Roza E, Vladacenco OA, Teleanu DM, Neacsu R, Teleanu RI (2023). Functional neurological disorder-old problem new perspective. Int J Environ Res Public Health.

[38] Lopez MR, LaFrance WC (2022). Treatment of psychogenic nonepileptic seizures. Curr Neurol Neurosci Rep.

[39] Nicholson TR, Voon V (2016). Transcranial magnetic stimulation and sedation as treatment for functional neurologic disorders. Handbook of Clinical Neurology.

[40] Löwe B, Gerloff C (2018). Functional somatic symptoms across cultures: perceptual and health care issues. Psychosom Med.

[41] Arbabi M, Masjedi N, Eybpoosh S, Shafaghi L, Yasami M (2022). Pathway to care of patients with functional neurological disorders in Iran. J Neurol Neurosurg Psychiatry.

[42] Tayeb H, Khayat A, Milyani H, Alsawwaf Y, Alzaben F, Koenig HG (2018). Supernatural explanations of neurological and psychiatric disorders among health care professionals at an academic tertiary care hospital in Saudi Arabia. J Nerv Ment Dis.

[43] Tayeb HO, Alsawwaf Y, Kokandi S (2023). Mind-body dualism and medical student attitudes toward mental illness in Saudi Arabia. Int J Psychiatry Med.

[44] Glannon W (2020). Mind-brain dualism in psychiatry: ethical implications. Front Psychiatry.

[45] Aftab M (2005). Is Islam committed to dualism in the context of the problem of free will?. J Cogn Neuroethics.

[46] Lebowitz MS, Appelbaum PS (2019). Biomedical explanations of psychopathology and their implications for attitudes and beliefs about mental disorders. Annu Rev Clin Psychol.

[47] Baloch A (2021). Functional Neurological Disorder: A Faith and Cultural Perspective. https://www.proquest.com/dissertations-theses/functional-neurological-disorder-faith-cultural/docview/2617310045/se-2.

[48] Alqassas M, Alatiyah MH, Aldharman SS, Alburayman MZ, Alrashed MH, Al-Sultan AA, Alrahil R (2022). Approach and clinical practice of functional movement disorders among neurologists in Saudi Arabia. Cureus.

[49] Kanemoto K, LaFrance WC Jr, Duncan R (2017). PNES around the world: where we are now and how we can close the diagnosis and treatment gaps-an ILAE PNES Task Force report. Epilepsia Open.

